# Risk factors for a failed trial without catheter following convective water vapor thermal therapy (CWVTT‐Rezum)

**DOI:** 10.1111/luts.12483

**Published:** 2023-05-26

**Authors:** Michael D. Felice, Kaylin Kim, Sarang Janakiraman, Gaurav Pahouja, William Adams, Erin Fruth, Ahmer Farooq, Kevin T. McVary

**Affiliations:** ^1^ Stritch School of Medicine Loyola University Medical Center Maywood Illinois USA; ^2^ Center for Male Health Loyola University Medical Center Maywood Illinois USA

**Keywords:** minimally invasive surgical procedures, prostate, prostatic hyperplasia, urinary catheters, urinary retention

## Abstract

**Objectives:**

Convective water vapor thermal therapy (CWVTT‐Rezum) is a minimally invasive surgical therapy that is being increasingly utilized for bladder outlet obstruction. Most patients leave the site of care with a Foley catheter in place for a mean reported duration of 3–4 days. A minority of men will fail their trial without catheter (TWOC). We aim to identify the frequency of TWOC failure following CWVTT and its associated risk factors.

**Methods:**

Patients who underwent CWVTT at a single institution from October 2018 to May 2021 were retrospectively identified and pertinent data extracted. The primary endpoint was TWOC failure. Descriptive statistics were performed, and rate of TWOC failure was determined. Potential risk factors for failed TWOC were assessed through univariate and multivariate logistic regression.

**Results:**

A total of 119 patients were analyzed. Seventeen percent (20/119) had a failed TWOC on their first attempt. Of those, 60% (12/20) failed in a delayed fashion. In patients who failed, the median number of total TWOC attempts required for success was two (interquartile range [IQR] = 2–3). All patients eventually had a successful TWOC. The median preoperative postvoid residual for successful and failed TWOC was 56 mL (IQR = 15–125) and 87 mL (IQR = 25–367), respectively. Preoperative elevated postvoid residual (unadjusted odds ratio [OR] 1.02, 95% CI: 1.01–1.04; adjusted OR 1.02, 95% CI: 1.01–1.04) was associated with TWOC failure.

**Conclusions:**

Seventeen percent of patients failed their initial TWOC after CWVTT. Elevated postvoid residual was associated with TWOC failure.

## 1 INTRODUCTION

The prevalence of benign prostatic hyperplasia (BPH) rises drastically as age advances. Between 50% and 70% of men suffer from lower urinary tract symptoms (LUTS) associated with BPH after the age of 50 years. Furthermore, evidence suggests that the prevalence of LUTS/BPH is as high as 80% by the eighth decade.^1^ Transurethral resection of the prostate (TURP) has traditionally been considered the gold standard surgical treatment option.^2^ While TURP has demonstrated its efficacy in improving urinary symptoms, acute safety concerns and long‐term negative impacts such as erectile and ejaculatory dysfunction, possible incontinence, as well as other associated complications have been well documented.^3^ As an alternative to TURP, minimally invasive surgical treatments (MIST) have emerged as options to relieve symptoms while minimizing or eliminating hospital stays and reducing complications. Convective water vapor thermal therapy (CWVTT‐Rezum) (Rezum System, Boston Scientific, Marlborough, Massachusetts) is an innovative MIST cleared by the United States Food and Drug Administration (US FDA) in 2015 to reduce prostate tissue volume associated with BPH, including hyperplasia of the central zone and/or a middle lobe. This therapy transfers stored thermal energy (540 calories/mL H_2_O) as vapor to the prostatic tissue.^4^ No thermal effects occur outside the targeted treatment zone. This addresses the limitation of conductive heat transfer experienced with other MIST, such as transurethral needle ablation (TUNA) and transurethral microwave therapy (TUMT), where cell kill gradient was noted to be a limiting factor in outcomes.^5^ CWVTT can treat difficult anatomic variants like obstructive middle lobes without advanced training or techniques. CWVTT challenges the long‐held algorithm of men needing endless medications followed by an invasive surgery if their LUTS/BPH progresses.

CWVTT is being increasingly utilized as the primary surgical treatment for bladder outlet obstruction for LUTS/BPH. Most patients will leave the site of care (office or ambulatory surgical center) with a Foley catheter in place. Catheter duration is reported in the literature as a mean of 3.4 days.^6^ However, absent of any clinical trial evidence supporting this practice, this choice is per provider preference and guided only by anecdotal evidence. A minority of treated men will fail their trial without catheter (TWOC) either at time of catheter removal or re‐present with delayed acute urinary retention (AUR).^7^ This variation in failed TWOC is worrisome for both patients and caregivers. A better understanding of the factors that drive a failed TWOC is needed. In this pilot study, we aim to identify risk factors for TWOC failure that may improve provider decisions and patient counseling on post‐Rezum bladder management.

## 2 METHODS

Patients ≥18 years of age who underwent CWVTT at Loyola University Medical Center, from October 2018 to May 2021, were included for analysis. Patients with confirmed neurogenic bladder on urodynamic testing, preoperative catheter‐dependent urinary retention, and postoperative urinary tract infections, as defined by a positive urine culture with associated symptoms, were excluded. These patients were retrospectively identified by Current Procedural Terminology Codes 53852 and 53854. Patient consent requirement was waived given the retrospective nature and patient de‐identification. Patient, operative, and outcome data were extracted and stored on a Health Insurance Portability and Accountability Act (HIPPA)‐compliant database (REDCap).^8^ The primary endpoint was TWOC failure. The primary aim of the study was to identify risk factors for TWOC failure following CWVTT. Secondary aims included identifying the frequency of TWOC failure and comparing the frequency of acute care visits between those with TWOC success and failure.

Preoperative prostate imaging was performed with either cross‐sectional imaging (computed tomography or magnetic resonance imaging) or transrectal ultrasound. Prostate volume was calculated by $V=l\cdot w\cdot h\cdot (\frac{1}{6}\pi$). Preoperative urodynamics consisted of both pressure flow studies and uroflowmetry. Peak urinary flow rate (Qmax) was measured by pressure flow studies, and postvoid residual (PVR) was measured following uroflowmetry with a transabdominal ultrasound bladder scanner. Bladder outlet obstruction index (BOOI) was calculated by BOOI = detrusor pressure_Qmax_ − (2)Qmax. Bladder contractility index (BCI) was calculated by BCI = detrusor pressure_Qmax_ + (5)Qmax. Bladder voiding efficiency (BCE) was calculated by BCE = $\left(\frac{\mathrm{PVR}}{\text{bladder capacity}}\right)\cdot 100\%.$ Detrusor function (i.e., normal detrusor function, detrusor overactivity, and detrusor underactivity) was based on the reading physician's interpretation which was included in the final urodynamics report.

Consistent with pivotal trial approaches, the number of treatments were based on prostate volume and visual inspection of the prostatic urethra, and further balanced with the patient's interest in preserving ejaculatory function. Postoperative catheter duration was not standardized and was per provider discretion and influenced by the day of the week the treatment was performed (i.e., if CWVTT performed on a Friday, the catheter was typically removed the following Monday) and clinic availability. All patients were continued on their preoperative LUTS/BPH medications through their TWOC, with the majority of men discontinuing these mediations by 3 months postoperatively. Failed TWOC was defined as the inability to void at all or rising PVR despite multiple attempts at bladder emptying. Immediate TWOC failure was defined as failure to urinate immediately following catheter removal. Delayed TWOC failure was defined as subsequent AUR after otherwise documented successful voiding. Some patients with TWOC failure were offered clean intermittent catheterization (CIC) at the provider's discretion. The date of subsequent TWOC success in patients performing CIC was defined as the date of documentation that the patient had stopped CIC. Patients taking anticholinergics or mirabegron at time of initial TWOC failure were instructed to stop these medications. All patients were included, regardless of missing data, unless explicitly excluded by the exclusion criteria.

SAS version 9.4 (Cary, North Carolina) was used for statistical analysis. Baseline patient characteristics were reported as fractions with percentages or medians with interquartile ranges (IQR) as appropriate. The exact logistic regression model was used for estimating unadjusted and adjusted odds of TWOC failure.^9^ The number of explanatory variables included in the multivariable model was determined by event rate and model fit statistics (Akaike information criterion), such that inclusion (or exclusion) of a covariate optimized the precision of explanatory variables included in the model.^10^, ^11^ For all quantitative explanatory variables, the linearity assumption was retained using a Hosmer and Lemeshow goodness‐of‐fit test.

The logistic regression model was used to create Figure 2. The model

$$\hat{\pi }=\frac{e^{{\hat{\beta }}_{0}+{\hat{\beta }}_{1}\left(\mathrm{PVR}\right)}}{1+e^{{\hat{\beta }}_{0}+{\hat{\beta }}_{1}\left(\mathrm{PVR}\right)}}=\frac{e^{-2.12+0.004\left(\mathrm{PVR}\right)}}{1+e^{-2.12+0.004\left(\mathrm{PVR}\right)}}$$

predicts the probability of TWOC failure ($\hat{\pi }$), which is a function of preoperative PVR. For any value of PVR, one can use the equation to find the probability of TWOC failure.

Complications were recorded according to the Clavien–Dindo classification. An exact Wilcoxon rank sum test was used to test the distribution of the number of acute care visits between those who succeeded and failed TWOC.

## 3 RESULTS

One hundred and nineteen patients qualified for analysis. All patients had a Foley catheter placed at the end of their procedure. Table 1 depicts baseline patient characteristics. Neurologic conditions included diabetic neuropathy, epilepsy, spinal stenosis, transient ischemic attacks without residual deficits, and gait and balance dysfunction of unknown etiology. Eight of 10 patients with neurologic conditions had urodynamic testing done, and none resulted in diagnosis of neurogenic bladder. Of the patients, 75.6% (90/119) and 93.3% (111/119) completed preoperative urodynamic testing and prostate volume imaging, respectively. Average prostate volume was 47.9 cm^3^ (IQR = 35.1–66.7 cm^3^). Median number of treatment sites for all patients was six (IQR = 5–8). Mean time to first TWOC was 4.0 days (SD = 1.7 days). Time to first TWOC was similar between successful (mean = 4.1 days, SD = 1.7 days) and failed TWOC (mean = 3.4 days, SD = 1.3 days; *p* = .11).

**TABLE 1:** Baseline patient characteristics.

	TWOC result			
	Success (*n* = 99)		Failure (*n* = 20)	
Variable	Median/*n*	IQR/%	Median/*n*	IQR/%
Age (years)	67	(61–73)	67.5	(61.5–72)
Ethnicity				
Caucasian	88/99	88.9%	18/20	90.0%
Asian	2/99	3.0%	1/20	5.0%
African American	3/99	2.0%	0/20	0.0%
Hispanic/Latino	4/99	4.0%	1/20	5.0%
Unknown	2/99	2.0%	0/20	0.0%
BMI	28.9	(25.7–31.1)	30	(26.7–33.25)
Comorbidities				
Diabetes	16/99	16.2%	5/20	25.0%
Hypertension	52/99	52.5%	7/20	35.0%
Neurologic conditions	9/99	9.1%	1/20	5.0%
Preoperative composite IPSS	19	(16–23)	24	(18–27)
Preoperative IPPS‐QOL	5	(4–5)	5	(4–6)
Prostate volume (cm^3^)	47.9	(36.1–67.8)	47.9	(31.4–58.1)
Urodynamics				
Pre‐op PVR (mL)*	56	(15–125)	87	(25–367)
Bladder capacity (mL)	319.5	(189.5–450.5)	346	(214–542)
Detrusor function				
Underactive	1/69	1.5%	2/18	11.1%
Normal	30/69	43.5%	8/18	44.4%
Overactive	38/69	55.1%	8/18	44.4%
Qmax (mL/s)	7.2	(4.2–9.5)	7.8	(3.2–12.2)
BOOI	48	(29.4–72)	49.1	(27.8–80.3)
Bladder voiding efficiency	82.60%	(66.2%–94.2%)	67.80%	(29.7%–92.4%)
Bladder contractility index	103.1	(81.6–131.9)	99.6	(85–127)
Medications				
Alpha‐blocker	76/99	76.8%	18/20	90.0%
5‐ARI	28/99	28.6%	4/20	20.0%
Anticholinergic or mirabegron	10/99	6.1%	4/20	20.0%
Tadalafil	4/99	4.0%	0/20	0.0%
Prior bladder outlet procedure	4/99	4.0%	2/20	10.0%
Total number of treatments	6	(5–8)	5	(5–8)
Total number of treatments to middle lobe/median bar	1	(1–2)	1	(1–1)

*Note*: Values are presented as a fraction with percentages or medians with IQR as appropriate.

Abbreviations: 5‐ARI, 5‐alpha‐reductase inhibitor; BMI, body mass index; BOOI, bladder outlet obstruction index; IPSS, International Prostate Symptom Score; IPSS‐QOL, International Prostate Symptom Score‐Quality of Life; IQR, interquartile range; pre‐op PVR, preoperative postvoid residual; Qmax, peak urinary flow rate; TWOC, trial without catheter.

* Denotes significant difference between TWOC success and failure on adjusted analysis.

**FIGURE 1:**
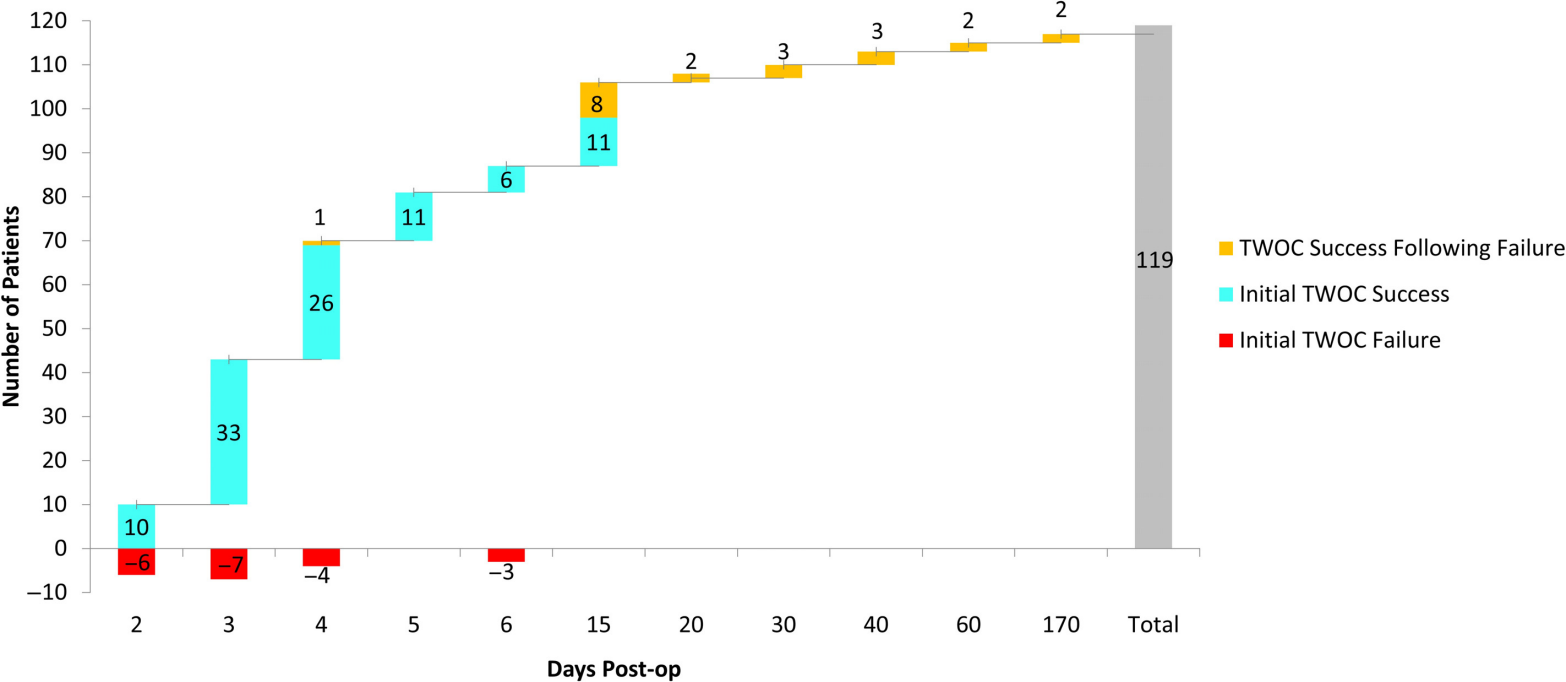
Trial without catheter outcomes based on timing. A total of 117/119 (98%) patients had a documented date of trial without catheter; 20/20 (100%) of patients who failed initial trial without catheter ultimately became catheter independent. TWOC, trial without catheter.

**FIGURE 2:**
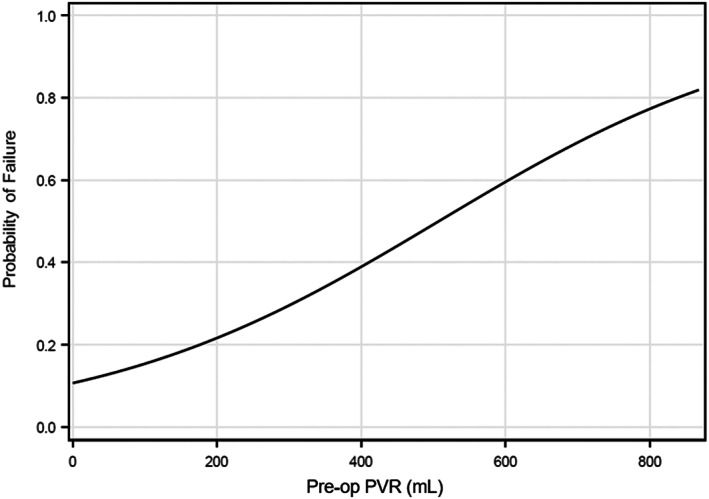
Probability of trial without catheter (TWOC) failure as a function of preoperative postvoid residual (mL) based on adjusted logistic regression model (Table 2). The model $\hat{\pi }=\frac{e^{{\hat{\beta }}_{0}+{\hat{\beta }}_{1}\left(\mathrm{PVR}\right)}}{1+e^{{\hat{\beta }}_{0}+{\hat{\beta }}_{1}\left(\mathrm{PVR}\right)}}=\frac{e^{-2.12+0.004\;\left(\mathrm{PVR}\right)}}{1+e^{-2.12+0.004\;\left(\mathrm{PVR}\right)}}$ states that the probability of TWOC failure ($\hat{\pi }$) is a function of increasing preoperative PVR. For any PVR value, one can use the equation to find the probability of TWOC failure. The risk of failed TWOC increased by ~2% with each 5‐mL increase in preoperative postvoid residual. PVR, postvoid residual.

Seventeen percent (20/119) of patients failed their initial TWOC (Figure 1). Of these men, 60% (12/20) failed their TWOC in a delayed fashion (median = 2 days, IQR = 0–3). All patients ultimately became catheter independent (Figure 1). In patients who failed, the median total number of TWOC attempts (including initial TWOC failure) required for success was two (IQR = 2–3). Three patients demonstrated detrusor underactivity on urodynamic testing, of which two failed their initial TWOC. The median preoperative PVR for successful and failed TWOC was 56 mL (IQR = 15–125) and 87 mL (IQR = 25–367), respectively. The median postoperative PVR for patients with successful TWOC was 45 mL (IQR = 11–86 mL). Table 2 depicts odds of TWOC failure. Controlling for body mass index and preoperative total International Prostate Symptom Score (IPSS), every 5‐mL increase in preoperative PVR increased the odds of TWOC failure by approximately 2% (adjusted odds ratio 1.02, 95% CI: 1.01–1.04; *p* = .01). Figure 2 demonstrates that the probability of TWOC failure (y‐axis) increases as the preoperative PVR (x‐axis) increases. Figure 2 can be used to estimate the probability of failure for any given preoperative PVR value. For example, at a preoperative PVR value of 530, the probability of failure is 50%.

**TABLE 2:** Odds of TWOC failure. Univariable and multivariable logistic regression models predicting odds of TWOC failure.

	Valid *n*	Unadjusted		Adjusted	
		Odds ratio (95% CI)	*p*	Odds ratio (95% CI)	*p*
Number of total treatments (per count)	119	0.99 (0.80–1.21)	.93		
Number of treatments to middle lobe/median bar (per count)	110	0.52 (0.09–1.83)	.44		
Prostate volume (per cm^3^)	111	0.99 (0.96–1.01)	.44		
Preoperative PVR (per 5‐mL increase)	109	1.02 (1.01–1.04)	.01	1.02 (1.01–1.04)	.01
Bladder capacity (per 5‐mL increase)	86	1.00 (0.99–1.01)	.57		
Qmax (per mL/s)	86	0.98 (0.87–1.08)	.72		
BOOI (per 1‐unit increase)	81	1.00 (0.98–1.02)	.90		
Age (per year increase)	118	1.00 (0.95–1.06)	.99		
BMI (per 5‐kg/m^2^ increase)	118	1.27 (0.78–2.06)	.34	1.44 (0.81–2.57)	.21
Diabetes: Yes vs. No	119	1.72 (0.43–5.96)	.51		
Hypertension: Yes vs. No	119	0.49 (0.15–1.45)	.24		
Neurological condition: Yes vs. No	119	0.53 (0.01–4.23)	.94		
Race: Non‐White vs. White	117	1.09 (0.11–5.92)	.99		
Detrusor overactivity: Yes vs. No	87	0.66 (0.20–2.10)	.59		
Alpha‐blocker: Yes vs. No	119	2.71 (0.58–25.79)	.30		
5‐ARI: Yes vs. No	117	0.67 (0.15–2.35)	.72		
Anticholinergic or mirabegron: Yes vs. No	119	2.21 (0.45–8.93)	.37		
Tadalafil: Yes vs. No	118	0.62 (0.00–3.41)	.34		
Prior bladder outlet procedure: Yes vs. No	119	2.61 (0.22–19.85)	.53		
Bladder voiding efficiency (per 5% increase)	86	0.92 (0.84–1.01)	.08		
Bladder contractility index (per 5‐unit increase)	80	0.98 (0.91–1.01)	.64		
Preoperative composite IPSS (per point increase)	98	1.10 (1.01–1.20)	.03	1.08 (0.98–1.18)	.12
Preoperative IPSS‐QOL (per point increase)	98	1.04 (0.83–1.29)	.55		

*Note*: Controlling for body mass index and preoperative total IPSS, every 5‐mL increase in preoperative PVR increased the odds of TWOC failure by ~2%. ‘Valid *n*’ is the number of patients used to compute the unadjusted estimates. The number of patients used to compute the adjusted estimates was 94 (with 19 events). Confidence limits and significance values are from the exact logistic regression model.

Abbreviations: 5‐ARI, 5‐alpha‐reductase inhibitor; BMI, body mass index; BOOI, bladder outlet obstruction index; IPSS, International Prostate Symptom Score; IPSS‐QOL, International Prostate Symptom Score‐Quality of Life; PVR, postvoid residual; Qmax, peak urinary flow rate.

There were no Clavien–Dindo grade ≥3 postoperative complications. However, the distribution of acute care visits was higher among those who failed TWOC (*M* = 1.84, SD = 0.76) than those who succeeded (*M* = 1.29, SD = 0.70; exact *p* < .001). Of 30 patients with a postoperative acute care visit, LUTS secondary to AUR following initial successful TWOC (i.e., delayed TWOC failure) was the most common complaint (63.3%, *n* = 19). Other complaints included hematuria (13.3%; 4/30), worsening irritative LUTS (13.3%; 4/30), Foley catheter‐related issues (16.7%; 5/30—i.e., drainage around Foley catheter, nondraining Foley catheter), and benign chest pain (3.3%; 1/30).

## 4 DISCUSSION

Recently published 5‐year data reporting on the outcome of CWVTT showed significant improvement of LUTS at ≤3 months post thermal therapy, remaining durable through 5 years (IPSS reduced 48%, quality of life increased 45%, Qmax improved 44%, benign prostatic hyperplasia impact index decreased 48%). Surgical retreatment rate was 4.4% with no reports of device‐related or procedure‐related sexual dysfunction or sustained de novo erectile dysfunction.^6^, ^12^ Initially, postoperative catheter placement was at the provider's discretion, with up to half of postoperative catheter placements due to failure to adequately void post procedure. In the pivotal trial and in current urology practice, most patients will have a Foley catheter placed at the conclusion of their procedure. Reported median catheter time is ~3–6 days in prior studies.^4^, ^6^ However, catheter durations were chosen empirically. There is currently no scientific evidence to guide catheter duration nor insight into what factors drive TWOC failure in some men. This is the first study to evaluate risk factors of TWOC failure.

The incidence of TWOC failure in this cohort was higher than expected based on prior reports of postoperative AUR at any time point. Urinary retention rates were initially high while the procedure was being developed. Dixon et al. (2015) reported AUR in approximately one‐third of patients.^4^ However, the incident of AUR in subsequent cohorts was ~4%–6%, which more closely resembles our current technique.

Interestingly, 37.5% (6/16) and 15.7% (11/70) of patients failed their initial TWOC on postoperative Day 2 and Days 3/4, respectively. Beyond 4 days, 9.7% (3/31) patients failed their initial TWOC, with all failures occurring on Day 6. Two of these patients failed in a delayed fashion, both within 48 h with PVR > 300 mL. Notably, preoperative urodynamic testing was consistent with obstruction in the setting of asymptomatically elevated PVR (350–575 mL) for these three patients. All three patients eventually passed their void trial, and their PVR have since normalized (PVR < 150 mL). These patients may have been destined to fail regardless of the initial TWOC timing. For most patients, waiting until postoperative Day 3 to perform the initial TWOC may improve success rates. However, further prospective research is needed to better validate this hypothesis.

Unique to this report, elevated preoperative PVR was associated with TWOC failure. The risk of failed TWOC increased ~2% with each 5‐mL increase in preoperative PVR. Given that an increased PVR likely reflects a preoperative bladder decompensation, a linear relationship between PVR volume and failure to void has an intuitive compatibility.^13^ A bladder scan is a cheap and noninvasive clinic‐based test. Despite the known variability and limitations of PVR, it is a readily available test and utilized in the preoperative evaluation of men with BPH/LUTS as recommended by the 2021 American Urological Association (AUA) BPH clinical guidelines.^2^ A preoperative PVR could help guide postoperative catheter duration in men undergoing Rezum. Men with elevated PVR, particularly those with PVR > 200, may benefit from longer postoperative catheter duration. Similarly, further prospective research is needed to better validate this hypothesis as well.

As expected, TWOC failure is associated with increased acute care visits. This highlights the importance of optimizing TWOC success to improve patient and provider satisfaction and decrease unnecessary healthcare costs.

This study has three main limitations. First, there was no standardized post‐op catheter duration protocol given the study's retrospective nature. Catheter duration was per provider discretion, clinic availability, and influenced by the day of the week the procedure was performed. For example, patients undergoing the procedure on Wednesday were scheduled for TWOC on Friday. Whereas patients whose procedure was on Friday were scheduled for TWOC the following Monday. Second, some patients on CIC self‐discontinued catheterization at home based on low catheterized postvoid volumes. CIC discontinuation, that is, TWOC success, may have been weeks to months prior to their follow‐up appointment where catheter discontinuation, and TWOC success date, was documented. Third, our sample size may have been underpowered, given the sparse number of TWOC failures, to detect a true significant difference among other possible risk factors. Specifically, diabetes, bladder capacity, percent voided volume, detrusor underactivity, bladder contractility index, and Qmax remain suspect as possible factors influencing TWOC failure. Regardless, these data can help providers better counsel patients on post‐CWVTT expectations and identify elevated PVR as a risk factor for TWOC which may aid the decision regarding postoperative catheter duration.

Seventeen percent of patients failed their initial TWOC after CWVTT. Elevated PVR was associated with TWOC failure. Failed TWOC was associated with increased acute care visits. Importantly, all patients eventually had a successful TWOC. Prospective research is needed to further delineate and confirm risk factors for failed TWOC. Furthermore, determining optimal timing of postoperative TWOC is paramount.

## AUTHOR CONTRIBUTIONS

All authors contributed to the study conception and design, data collection and/or analysis, and manuscript creation.

## FUNDING INFORMATION

This research was partialy supported by NIAID T35 AI125220.

## CONFLICT OF INTEREST STATEMENT

Michael D. Felice, Kaylin Kim, Sarang Janakiraman, Gaurav Pahouja, William Adams, Erin Fruth, and Ahmer Farooq: None. Kevin T. McVary: NIDDK (PI and consultant), ProDeon (PI and consultant), Boston Scientific (PI and consultant), Francis Medical (consultant), Urotronic (consultant).

## ETHICS APPROVAL

This research was approved by the Loyola University Medical Center Internal Review Board (IRB# 213863). Consent requirement was waived given the retrospective nature and patient de‐identification. The study was performed in accordance with the Declaration of Helsinki.

## Data Availability

The data that support the findings of this study are available on request from the corresponding author. The data are not publicly available due to privacy or ethical restrictions.
